# HIV Self-Testing: A Discussion on the Benefits, Limitations, and Implications for Public Health with a Focus on Poland

**DOI:** 10.3390/diagnostics14222475

**Published:** 2024-11-06

**Authors:** Aleksandra Kozieł, Igor Domański, Aleksandra Szymczak, Tomasz Dudzik, Brygida Knysz, Bartosz Szetela

**Affiliations:** 1 Department of Infectious Diseases, Liver Disease and Acquired Immune Deficiencies, Wroclaw Medical University, 50-367 Wroclaw, Poland; igor.domanski@student.umw.edu.pl (I.D.); aleksandra.szymczak@umw.edu.pl (A.S.); brygida.knysz@umw.edu.pl (B.K.); bartosz.szetela@umw.edu.pl (B.S.); 2 All Saint’s Clinic, Wrocławskie Centrum Zdrowia SP ZOZ, 50-136 Wroclaw, Poland

**Keywords:** HIV self-testing, HIV detection, Poland

## Abstract

Background/Objectives: A late HIV diagnosis represents a significant public health concern in Poland, with approximately 50% of patients being identified as late presenters (LPs), resulting in the delayed initiation of treatment. This study assesses the potential of HIV self-testing (HIVST) to enhance early detection, particularly among heterosexual individuals, and evaluates its advantages and limitations within the Polish context. Methods: This study examines the advantages and disadvantages of HIVST by analyzing data from various studies. It focuses on the acceptability, sensitivity, and specificity of HIVST, comparing blood-based and oral fluid-based tests. Moreover, the economic impact and potential public health benefits of HIVST in Poland are evaluated. Results: HIVST is well-accepted, especially among key populations; it can reduce stigma and enhance privacy. The results of studies conducted in Poland indicate that heterosexuals are more inclined to use self-testing methods than traditional diagnostic procedures. On the other hand, HIVST has the potential for false-negative results due to the serological window and the possibility of missed diagnoses of other sexually transmitted infections (STIs). Moreover, the cost of HIVST remains a significant barrier, as it is not publicly funded in Poland. Conclusions: Despite its limitations, HIVST offers a number of significant benefits, including increased rates of testing and earlier detection, which could prove vital in reducing the transmission of HIV in Poland. This study proposes that increased funding, the integration of HIVST into public health strategies, and further research to enhance its implementation, alongside education and support for its effective use, should be prioritized.

## 1. Introduction

In 2023, it was estimated that 5.4 million people globally were unaware that they were living with HIV, which accounts for approximately 14% of the total 39.9 million people infected worldwide [1]. However, this figure may be an underestimate. In countries with a poor healthcare infrastructure or where there is significant stigma surrounding HIV, the coverage of testing services is often much lower. Consequently, many individuals, particularly in regions such as sub-Saharan Africa and parts of Asia, may remain undiagnosed, which presents challenges for effective treatment and prevention efforts [2]. In numerous areas, accessibility to testing remains problematic and the persistent fear of stigma and discrimination poses a significant barrier to the widespread acceptance of HIV testing services globally [3].

**Figure 1:**
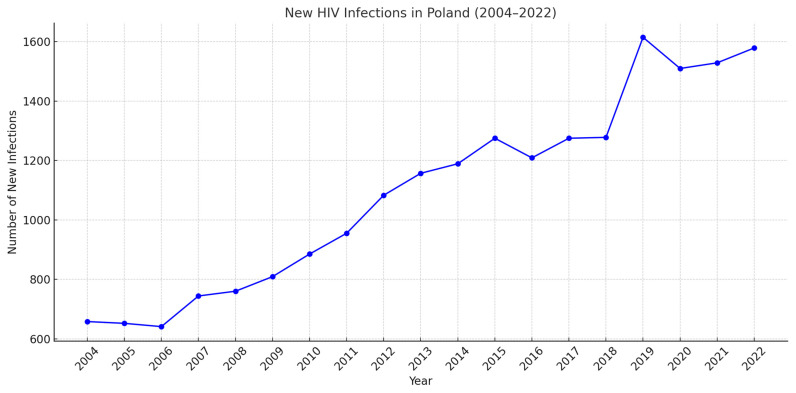
The number of new infections in Poland from 2004 to 2022 [4].

In Poland, since the initiation of HIV diagnostics, there has been an observable increase in the number of infections [4], with a peak occurring in 2019. In subsequent years, particularly between 2020 and 2021, the number of new cases decreased, largely due to the impact of the COVID-19 pandemic, which reduced access to testing and healthcare services. However, a recent upward trend has been noted again, partly due to an increased number of tests being performed and advancements in diagnostics [5]. Despite these improvements, HIV infection continues to pose a serious epidemiological challenge in Poland. The worsening epidemiological situation is particularly concerning, especially considering that the number of new infections worldwide fell by 54% between 1996 and 2021 [6] (Figure 1).

In Poland, late diagnosis (defined as a CD4+ count < 350 cells/µL or the presence of opportunistic diseases indicative of AIDS [7]) is also a significant problem among a large number of patients [8,9]. Studies analyzing the epidemiological situation in Poland indicate that approximately 50% of patients are late presenters [8,10]. A comparable situation is observed in the WHO European Region, where 50.6% of those diagnosed in 2022 were considered late presenters. In Western Europe, a lower rate of 46.2% was observed, whereas in Eastern Europe, the rate was higher at 55.1% [11]. This delay in diagnosis prevents the timely initiation of antiretroviral treatment, which is crucial for reducing the risk of transmission and improving patient prognosis. Ensuring early detection and treatment is essential for better health outcomes and controlling the spread of HIV [12,13].

The World Health Organization (WHO) in 2016 recommended HIVST for HIV diagnosis in people who refuse other means, including people at risk, men, and young people [14]. For years, this topic has caused controversy [15]. However, at the 12th International IAS Conference on HIV Science in 2023, the WHO was calling on countries to expand the use of HIVST and promote testing through sexual and social networks to increase testing coverage and improve the uptake of HIV prevention and treatment services [16]. The purpose of HIVST is to minimize the number of HIV-infected persons who would not otherwise undergo testing in healthcare facilities [17].

In recent years, home testing devices have grown in popularity, opening up new opportunities. However, it is still not a common method of HIV testing in Poland. The aim of this paper is to explain what home testing is, its advantages, and its potential to accelerate HIV diagnosis in Poland, especially among heterosexual people.

## 2. Background to HIV Testing

### 2.1. When Can HIV Be Detected?

**Figure 2:**
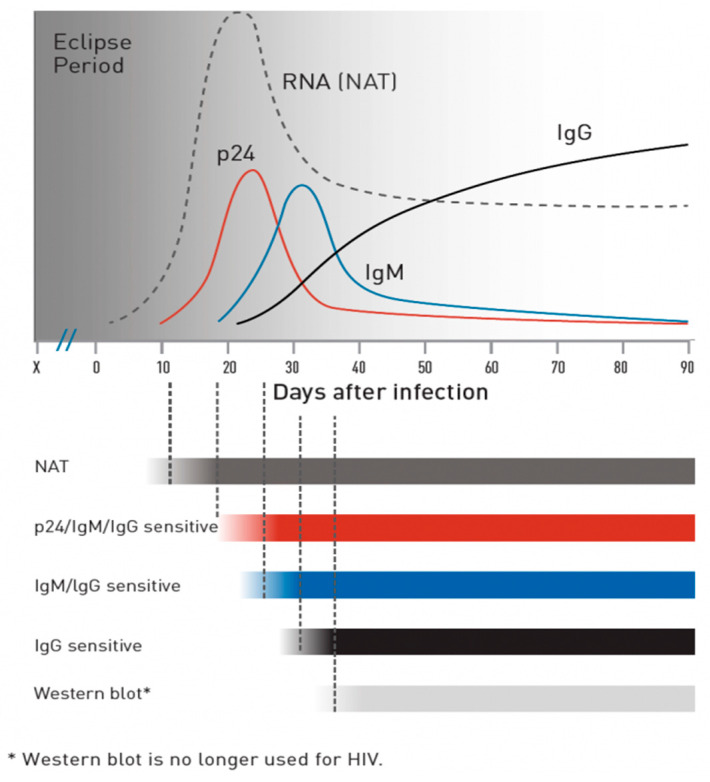
Source: CDC [24].

To initiate a discussion on HIV testing, it is crucial to first discuss the available types of tests and their reactivity time, which depends on the target being detected, the detectability of the target, and the concentration at which the test detects the target [18]. The period between infection and the detection of HIV is known as the eclipse period or window period [19,20]. The earliest indicator of HIV infection is the presence of HIV RNA in the blood. In half of the individuals infected, HIV RNA can be detected in plasma as early as 12 days after infection. RNA levels continue to rise until around day 20 to 30 of infection, after which they begin to drop but remain quantifiable [21]. The p24 protein is assayable from approximately day 15 after infection. Its level increases until day 25–30, but after about 50 days, its level becomes indeterminate again [22]. The presence of HIV triggers the production of IgM antibodies, which appear around day 20. IgG antibody levels rise around day 30–35, causing the subsidence of IgM antibody levels [23] (Figure 2).

### 2.2. What Types of HIV Tests Are Available?

#### 2.2.1. Third Generation (ELISA Test)

The foundation of serological screening for HIV infection is the use of enzyme immunoassays (ELISAs/EIAs) [25]. These screening tests detect the presence of anti-HIV-1, anti-HIV-2, and anti-HIV-0 antibodies in the serum, which are directed against the structural proteins of HIV. These tests are characterized by high sensitivity (>98% three months after infection) and specificity (99%). Third-generation ELISA tests detect anti-HIV antibodies in both IgM and IgG classes, typically yielding positive results within 3–12 weeks after exposure [26,27,28]. They are therefore not recommended for use as part of common laboratory screening diagnostics. They are used in the form of rapid cassette or strip tests. Capillary blood test results in such a test are obtained within 5–30 min [29,30].

#### 2.2.2. Fourth Generation

Generation IV tests are advanced screening ELISA/EIA methods that, in addition to detecting IgG and IgM antibodies, also identify the p24 antigen. This antigen can be detected before antibodies appear, allowing for the detection of HIV infection as early as 2 weeks after exposure. These tests are particularly useful in the early phase of infection, before antibodies are detectable, and in the later stages, when the viral load is very high and antibodies might not be detected by third-generation tests [29,30].

#### 2.2.3. Fifth Generation

**Figure 3:**
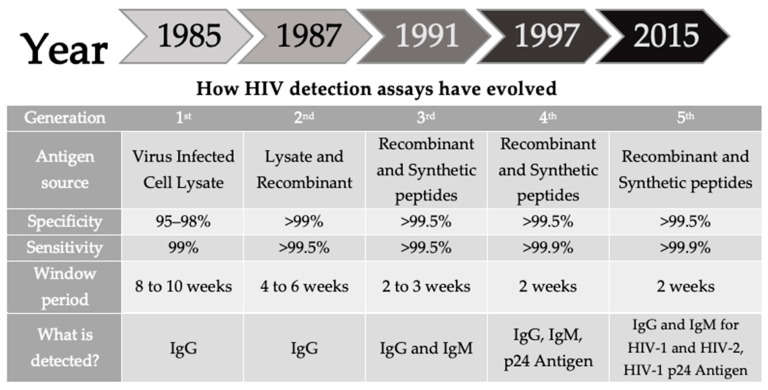
Schematic overview of the evolution of HIV detection tests, adapted from [26].

The fifth-generation methods detect both HIV antibodies and HIV-1 p24 antigens, and can differentiate between a positive result due to HIV-1 Abs, HIV-2 Abs, and HIV-1 p24 Ag, with separate results for each analyte [31] (Figure 3).

#### 2.2.4. HIV-1/HIV-2 Differentiation Test

After a positive screening test, an HIV-1/HIV-2 differentiation test is performed, according to European Guidelines [32]. This test works by detecting virus-specific antibodies in the patient’s blood. Antigens specific to HIV-1 and HIV-2 are used in the assay, binding to their corresponding antibodies if present. An enzyme is then added, which triggers a color change, indicating the presence of these antibodies. Differentiating between HIV-1 and HIV-2 is essential for selecting the most effective treatment plan, as the two types of HIV may require different therapeutic approaches [33].

#### 2.2.5. RNA Test/DNA Test (NAAT)

In the case of a positive screening test and a negative HIV-1/HIV-2 differentiation test, a molecular nucleic acid amplification test (NAAT) is performed. RNA tests detect the presence of the HIV virus and determine the viral load in a patient’s blood using PCR (polymerase chain reaction). The serological window for these tests’ ranges from 10 to 33 days after infection [34].

#### 2.2.6. WB/LIA

Until recently, Western blot (WB) and line immunoassay (LIA) tests have been used as confirmatory tests, but are now only recommended for the verification of infection in people already on antiretroviral treatment with an undetectable viral load or in HIV-infected people naturally controlling their infection with an undetectable viral load despite not taking antiretroviral treatment. These are serological tests that detect anti-HIV antibodies directed against more antigens than screening tests [29,30] (Figure 3).

## 3. HIV Self-Testing (HIVST)

An HIV self-test, also known as a rapid self-test for home use, is an antibody examination designed for private use. On the medical market, third-generation tests are usually available. These tests can be divided based on the specimen collection method. Some tests examine the presence of antibodies in blood, while others use oral mucosa exudate. Tests based on oral exudate require the collection of oral secretions with a spatula, which is then placed in a reagent tube. The blood-based tests involve pricking a fingertip, taking a drop of blood, which is introduced onto the test plate, and then adding the appropriate reagent. Results can be read after about 15–20 min [24]. Sensitivity and specificity, as provided by the manufacturers, are similar [17,35,36]. However, a meta-analysis comprising 25 articles indicates that blood-based self-tests consistently outperform oral fluid-based self-tests. Blood-based tests exhibit a sensitivity ranging from 96.2% to 100%, compared to 80% to 100% for oral fluid-based tests. Similarly, the specificity of blood-based tests ranges from 99.5% to 100%, whereas oral fluid-based tests range from 95.1% to 100% [17,37]. Despite this, surveys show that patients prefer to use oral tests [38]. Oral tests can give false-negative results if there is dryness of the mucosa, or if the patient has been chewing gum or smoking.

Both types of tests are interpreted in a similar way, with one line indicating a negative result, two lines indicating a positive result, and no line indicating that the test is faulty (no control line), similar to pregnancy tests.

In the realm of HIVST, there exist both supporters and opponents. In a forthcoming review paper, the advantages and disadvantages of disseminating HIVST will be outlined [39].

## 4. Advantages of HIVST

**HIVST is highly accepted, in particular by key populations** [39]. A review paper from 2013 analyzed eleven (two from sub-Saharan Africa, six from the US, two from Spain, and one from Singapore) studies in terms of the acceptability of HIV self-monitoring. Acceptability was measured as the percentage of individuals who were approached to take part in the study and ultimately underwent HIVST. A total of 70% of participants in the study agreed to self-test. The majority of participants in the studies belonged to key populations, including men who have sex with men (MSM), sexual partners of MSM, and individuals attending emergency departments or mobile testing units [40]. Unfortunately, little scientific research has been carried out on this subject in Europe. According to a 2012 French study, acceptability among the 377 participants was high, at 83% [41]. In another Spanish study, out of 267 participants, 78% agreed to take the self-test [42]. However, both studies mentioned showed no statistically significant difference between those who agreed to take the test and those who refused. There was no increase in interest in the test among key populations.

**HIVST diminishes social stigma and increases privacy** [39]. In an online survey conducted in Australia in 2009, out of over 2000 MSM respondents, 67.4% expressed their willingness to test more frequently if provided with a rapid self-test option. Factors independently associated with increased testing frequency with home self-testing in this study included, among others, a preference for more convenient testing, avoiding doctor visits for testing, and desiring immediate results [43]. A study in Botswana, based on collecting interviews with men, found that HIVST can overcome stigma. Men particularly appreciated the autonomy HIV self-testing afforded them in managing the testing and disclosure procedures. They valued the flexibility to self-administer tests at their convenience and location of choice, enabling them to navigate their emotions at their own pace and determine how and when to share the results with others. The ability to conduct HIVST privately, with sole knowledge of the test and its outcome, was especially appealing to men [44]. A 2015 meta-analysis based on an analysis of nine studies found that the likelihood of encountering any stigmatizing behavior within the community was reduced by 16% (RR = 0.84, 95% CI 0.79 to 0.89) among individuals who underwent home-based HIV counseling and testing (HCT) compared to those who did not. Among HIV-positive patients, the risk of facing any stigmatizing behavior decreased by 37% (RR 0.63, 95% CI 0.45 to 0.88) in the intervention group compared to the control group [45].

In addition to privacy, confidentiality should also be considered. The maintenance of confidentiality may not only reduce the risk of social stigma but also empower individuals to take control of their health without fear of breaching their privacy.

**HIV self-testing encourages reciprocal partner testing.** In an extensive review of 21 papers, which included 7.512 participants, it was revealed that when it comes to HIV self-testing, a significant majority of participants (80–97%) expressed readiness to also test their partners [46]. The 2023 meta-analysis, which included seven studies, found that the partner distribution of HIV self-testing kits increased the chance of testing a partner by 45% compared to a control group that used standard testing methods. The risk ratio (RR) was 1.45 and the confidence interval (95% CI) ranged from 1.05 to 2.02, indicating the statistical significance of this result [47]. Research conducted in Kenya on 300 women revealed that facilitating home-based testing not only promotes partner testing, but also fosters stronger emotional connections with partners, encourages more consistent condom usage, and enhances women’s autonomy in decision-making concerning their lives [48].

**HIVST is economically beneficial for the country.** A 2015 study, conducted in the United States, enlisted 2665 MSM with unknown or negative sero-status. Despite the considerable expenses tied to distribution, promotion, testing, and data collection, the outcomes were deemed favorable. Calculations indicated that 3.34 transmissions were averted, resulting in approximately USD 1.6 million dollars in savings on long-term antiretroviral treatment [49].

In contrast, research published in 2024 presents a study conducted in 2015 in Kenya. This study scrutinized the expenses linked with HIVST, juxtaposing the costs of distributing oral tests to truck drivers against those incurred when drivers underwent testing at local facilities. Self-testing emerged as nearly four times more costly. The escalated expenses were chiefly attributed to the cost of the testing kits themselves and the additional expense of telephone counseling provided by specially recruited personnel [50]. However, the study did not analyze the long-term costs connected to potential treatment.

**Figure 4:**
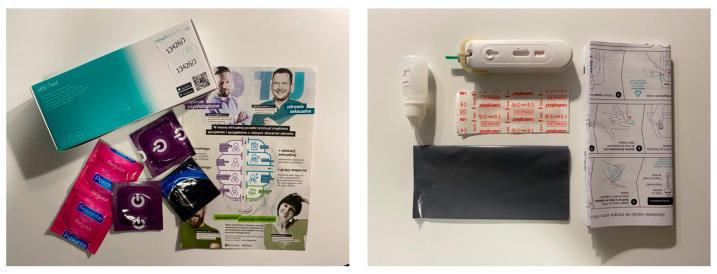
Home HIV test. Own work.

**HIVST increases the chances of detecting HIV infection in heterosexuals.** One of the studies, on the Polish population, was conducted at the turn of the year 2022/23 by the Wroclaw Health Center in cooperation with the website TyToTu.pl, where a link was provided allowing users to order a home HIV test. Users had to fill in shipping details and a short form. After a few days, the package was delivered to their homes by a courier. Inside was an HIV test along with instructions, informational leaflets, and condoms [51] (Figure 4).

Customers were asked to provide feedback after taking the test. In total, 1473 tests were sent out, and 887 pieces of feedback were received. Five people received a positive result (all heterosexual individuals), so the prevalence of HIV among the tested individuals was 0.34%, nearly 7 times higher than in the general population. Interestingly, the tests were most popular among heterosexual individuals (76.6%) [51], while among VCT users, MSM predominated [52]. This study showed that promotional campaigns can be highly popular and increase the chances of detecting infections. Furthermore, heterosexual individuals are encouraged to test, as they are often diagnosed at a more advanced clinical stage compared to MSM [9,53,54,55]. Another advantage of HIVST presented by the discussed study was the relatively high percentage of Ukrainians among the participants (9.77%), who face greater difficulties due to language barriers [51].

A similar study was conducted by the Foundation for Social Education (FES) in Poland during the COVID-19 pandemic, when access to medical facilities was limited. In total, 1062 tests were sent out. A total of 65.4% of the participants were HTX, while MSM accounted for 31.4%, which also demonstrates the significant popularity of this testing method among heterosexual individuals [56]. Heterosexual people, both men and women, are a group that requires attention and action to prevent infection and speed up diagnosis because of the high proportion of late diagnoses and the consequent poorer prognosis, compared with MSM [8,9,55,57,58]. The results from the cited studies are therefore a hope and an indication of how this group can be helped.

## 5. Disadvantages of HIVST

**HIVST has a high diagnostic value, but does not match the sensitivity and specificity of laboratory tests, because of the serological window and is associated with a risk of false-negative results.** A cross-sectional study conducted in Zambia in 2016–2017, which involved 2655 participants, demonstrated the high reliability of home HIV testing. Participants underwent a saliva-based HIV test at home, after a brief demonstration by a research assistant. Following the home test, participants visited a medical facility where qualified medical personnel administered the same test and drew blood for laboratory analysis. The results showed that the user-conducted user-read HIVST was 94.1% sensitive (95% CI: 90.2–96.7) and 99.7% specific (95% CI: 99.3–99.9) compared with the Zambian national RDT (rapid diagnostic test) algorithm (conducted by nurses), and 87.5% sensitive (95% CI: 82.7–91.3) and 99.7% specific (95% CI: 99.4–99.9) compared with the laboratory reference standard. Despite the reasonable sensitivity of HIVST when compared to the Zambian national rapid diagnostic test (RDT) algorithm, both HIVST and the RDT algorithm exhibited lower sensitivity in comparison to the laboratory reference standard [59]. As can be seen from the study cited, home tests are neither as sensitive nor as specific as laboratory tests. However, in a systematic review released in 2020, data from 10 randomized controlled trials (RCTs) involving 9679 participants were analyzed. The findings of the study indicated that there were no significant overall differences between HIVST and traditional laboratory tests (z = 1.17, *p* = 0.24) [60]. Paying attention to proper patient training in performing the test can increase the reliability of the test. Errors in technique on the part of the patient when undertaking the test may result in an inaccurate outcome [61]. Moreover, HIVST can result in problems maintaining compliance in people on antiretroviral treatment who have been tested despite no indication. In the research conducted in Khayelitsha, South Africa, 639 participants engaged in HIV self-testing. Among these, 401 records indicated a negative result, yet 5% of those individuals had previously been diagnosed with HIV and were under antiretroviral treatment. Clearer communication is necessary to reduce inappropriate testing, and upcoming HIV self-testing kits will feature a label reminding users not to utilize the test if they are taking antiretroviral drugs [62]. It is possible that an HIV self-test may be conducted during the serological window, a period when anti-HIV antibodies are not yet detectable in the blood. During this window period, the lack of detectable antibodies can result in a false-negative result [19,20]. It is of paramount importance to underscore the test’s diagnostic accuracy following the aforementioned window period, which typically extends for a duration of 2–12 weeks following potential exposure, with variations dependent on the individuals and on the manufacturer of the particular test [63,64,65]. It is imperative that this information be prominently displayed on the packaging of the test, in the accompanying leaflet, and in any instructional videos. Furthermore, this information should be conveyed by the pharmacist when the test is purchased. To ensure the accuracy of the results, it is recommended that the test be repeated after 3 months after risky behaviors [66]. It is also crucial to consider the potential for false-negative results, which can occur after the serological window. Despite the high sensitivity and specificity of the tests currently available for self-testing, there is a possibility of a false-negative result. Following the receipt of a negative result, the patient may feel reassured and may cease protection during intercourse, which could result in a more rapid spread of the virus [67].

**HIVST may be associated with a lack of medical consultation and thus failure to detect other potential sexually transmitted infections (STIs)** [60,68]. In many countries around the world, there are Voluntary Counselling and Testing (VCT) points where anyone can undergo free (or for a small fee) tests for HIV and other STIs. The use of HIVST may complicate the diagnosis of other STIs (i.e., syphilis, gonorrhea, and HCV). However, the literature presents different conclusions. In one randomized study conducted in Australia on a group of gay and bisexual men, no decrease in STI testing (at preferred facilities) was observed when using HIV self-testing (*p* = 0.276) [69]. Similar conclusions were drawn from a study conducted in the USA involving 65 gay men. The use of HIV self-testing did not affect the number of tests for other STIs (*p* > 0.05) [70]. However, importantly, in both studies, the tests were distributed in medical facilities, thus requiring contact with staff, which could have influenced the decision regarding testing for other sexually transmitted infections. A noticeable difference was observed in a study in the USA that consisted of 230 homosexual men, where some patients were provided HIVST in person at a medical facility and others were provided with it via mail. A significant difference and decrease in the number of tests performed for other STIs among the second group were observed (*p* = 0.0038) [71].

If an HIV self-test shows a positive result, it is essential that a confirmatory test be performed [24,72]. Clear information should be provided to the patient—such as on the packaging or in the accompanying leaflet—about the need to visit a medical facility where this test can be carried out. In most countries, the confirmatory test is free of charge, ensuring that the patient does not face any additional costs. Proper guidance and access to healthcare professionals are vital to ensure the accurate interpretation and follow-up of a positive result.

**HIVST may increase the risk of undertaking risky behavior**. A study conducted in China in 2024, involving 327 homosexual men, showed interesting findings. After self-testing for HIV, there was a statistically significant increase in the frequency of risky behaviors being undertaken by the participants. It showed an increase in the frequency of drug use (20.7% vs. 21.5%), commercial sex (14.6% vs. 17.3%), and unprotected anal sex (95.9% vs. 96.6%) [67]. The study found that a negative test result reduced participants’ awareness, contributing to risky behaviors. More research is needed on this subject, especially in Europe.

**HIVST may involve the patient’s cost of purchasing the test.** Some countries provide public funding for HIV self-testing as part of their public health programs. In Europe, countries such as France, the United Kingdom, Spain, and Portugal offer such support [73]. This enables individuals in these nations to access HIVST with greater ease, without having to bear significant out-of-pocket expenses. The cost of self-testing kits in Europe typically ranges between EUR 20 and EUR 35, with significant variation between countries [73]. This expense presents a substantial barrier for the typical resident. In some countries, free tests are only available through pilot programs, promotional campaigns, or research studies [73].

In Poland, the tests are not reimbursed by the national health system and are not a part of the national health policy. They are primarily available for private purchase online, at a cost to the purchaser [73]. Costs vary depending on the manufacturer and the type of test. Oral tests are generally more expensive than fingerpicks tests [38,73]. Research indicates that increasing the availability of free HIVST kits not only leads to a higher rate of testing, but also improves the detection of new infections, thereby enhancing public health outcomes [60,74].

## 6. Conclusions

HIVST is not ideal and has many limitations. However, despite its drawbacks, it has many advantages that could increase the number of tests performed, speed up diagnosis, and limit transmission. The risk/benefit ratio indicates that the commercialization of HIV self-tests is a favorable proposition. Despite limitations, home tests offer numerous benefits. The question arises of how to distribute tests and where to find funding for them. This paper serves as an appeal to the authorities in Poland to increase funding for initiatives related to HIVST. Test ordering could take place through specially created websites (promoted via social media). Proper test execution is crucial for obtaining reliable results. To achieve this, both the website and the test itself (e.g., in the form of a QR code) could include a link to an instructional video. Studies have shown that instructional videos/images are more easily absorbed by users than written instructions [75,76]. It should be emphasized repeatedly—both on the website, in the instructional video, and on the leaflet—to urgently schedule a doctor’s appointment in the case of a positive test result (websites should indicate precise locations, e.g., VCTs on the map of Poland). It is also important to inform customers and remind them of the limitations of tests related to the serological window. Moreover, in Poland, HIVST is already available in some pharmacies. Apart from offering the option for purchase, it is crucial for pharmacists to also offer concise guidance to customers on administering the test and providing counsel for individuals who return to the pharmacy with a positive result after purchasing a test there. Another possibility to increase the number of tests performed is to distribute them in family medicine or gynecology offices. The spread of HIVST in these medical practices would be an opportunity for doctors to offer the test right away if they suspect a patient is infected. This, of course, requires a proper conversation with the patient and advice on further treatment. Due to the small number of studies on HIVST carried out in Poland, a further analysis of the benefits of disseminating HIVST is needed.

## Data Availability

No new data were created or analyzed in this study. Data sharing is not applicable to this article.
